# Career Termination of Portuguese Elite Football Players: Comparison between the Last Three Decades

**DOI:** 10.3390/sports6040155

**Published:** 2018-11-28

**Authors:** António Carapinheira, Pedro Mendes, Pedro Guedes Carvalho, Miquel Torregrossa, Bruno Travassos

**Affiliations:** 1 Department of Sport Sciences, Universidade da Beira Interior, 6201-001 Covilhã, Portugal; acarapinheira@gmail.com; 2 Instituto Português de Administração e Marketing (IPAM), 1500-210 Lisboa, Portugal; pmendes@universidadeeuropeia.pt; 3 Instituto Superior da Maia (ISMAI), 4475-690 Maia, Portugal; pedro.guedes.carvalho@gmail.com; 4 CIDESD, Research Center in Sports Sciences, Health Sciences and Human Development, 5001-801 Vila Real, Portugal; 5 Facultat Psicologia, Autonomous University of Barcelona, 08193 Barcelona, Spain; miquel.torregrossa@uab.cat

**Keywords:** sport career, post-career, career termination, ecological model of human development

## Abstract

The aim of this study was to explore the process of career termination of elite soccer players, comparing the quality and the resources to support career termination over the last three decades. To this end, was developed a questionnaire defined by four sections: (a) biographical data, (b) athletic career, (c) quality of career termination and (d) available resources at the moment of career termination. Ninety male former elite Portuguese soccer players participated in this study. The results highlighted a decrease in the length of athletic career as football players and an increase in the number of years as youth players over the last 30 years. The results also revealed that the quality of career termination was difficult. The analysis of resources for career termination revealed an increase in a high level of education over the years. Despite the evolution in the level athletes’ education in the last three decades, the athletic career termination remained difficult and it was reported that they did not plan their career termination. In line with previous studies, the results highlight that the lack of plans for career termination is one of the most important factors that constrain the quality of career transition.

## 1. Introduction

In the last five decades, research in the area of sport career termination has advanced significantly with an improvement in the understanding of the factors associated with this process [1]. In fact, most studies in different sports have emphasized that 15 to 20% of retired athletes go through transitional difficulties, often requiring intervention and psychological support [2,3,4]. It can be explained by the multifactorial life process that constraints the process of career termination. That is, career termination is an individual process that is influenced by a range of individual, cultural and contextual causal factors that impacts on the process of career termination and on the adjustment of former players to the new life [2,5,6]. Thus, there is a need to understand the role of each factor in career termination of different countries or sports to really understand the impact of such process in athletes life [1].

Indeed, career termination is influenced by the situation, the self, the support and the individual strategies and it requires the adjustment of former athletes to the new psychological, social, familiar, occupational, and even financial status [7,8]. Previous research has shown that the reasons for the end of athletic career are generally: age, deselection, injuries and free choice [7], and these can be decisive in the adaptation to the post-sport phase [1,3]. However, different consequences can emerge according to the type of reasons pointed for career termination [6,9]. While free choice is usually associated with easy and healthy processes of career termination, deselection and injuries are usually associated with involuntary and difficult processes of career termination with correspondent negative emotions for the general of former players. The age is a more complex factor. Previous research reported that age can promote more easily or difficult transitions according to the level of voluntariness to abandon the athletic career [6]. According to that, Stambulova, Alfermann, Statler and Côté [1] pointed that the quality of a career termination depends of a multiplicity of causes, namely the crucial contribution of voluntary decision making, the importance of a planning for post-career life or the existence of social and individual resources for the athlete.

Also, over the last years it was emphasized that some cross-cultural research is necessary due to the diversity and needs of players of different countries, or even sports [5,10]. Supported by the Ecological Model of Human Development [11] it was advocated that research and intervention in career termination should avoid replicating general assumptions from any study in a certain country and sport to other particular context [10,12]. It was argued that the focus of research should be redirected towards the career termination of athletic career in a more holistic perspective and considering different levels of analysis [13]. Also, previous research suggested that research into career termination in sport should include the analysis of demographic characteristics, culture and sporting context, as well as the influence of sport systems and environmental contexts to really understand the process and the factors that constraint players development and career termination [7,14,15]. 

The ecological model of human development considers that human development is constraint by three different microsystems in interaction. The microsystem as the unit of the system is related with the social roles, interpersonal relations and experiences developed by an individual in a particular setting (e.g., family, club, team, the coach, the type of training tasks, the facilities of the club). The mesosystem comprises the relationship between two subsystems (e.g., family, club, school) and can be related with the social activities, the community support and the associated values shared and developed between participants. At the end, the macrosystem can be described as the social-cultural context (e.g., the national sport culture) that surrounds the development of the individual identity [16]. 

Aligned with the ecological model of human development, some research has been developed at macro-social level to understand the impact of the sociocultural context or even the national sport culture of each country in career termination of athletes. For example in a wide perspective, Stambulova, Alfermann, Statler and Côté [1] sustain that there are similarities between the North American, West European, and Australia traditions in career termination due to the resemblance between the social-cultural contexts of the respective countries. Focused in the European analysis, the European perspectives on athletic career termination project analyzed the process of athletes career termination in five different countries: Germany, Lithuania, Russia, France, and Sweden [3,10]. The results revealed an influence of the social-cultural context in the career termination process. For instance, West European athletes planned career termination in advance in comparison with East European athletes. Otherwise, in Russia there is a cooperation between the sport and scholar system promoting the development of a dual career and high level of formal education than in countries such as Germany, France or Sweden. Also, the athletes from Sweden reported high quality of transition than German, French or Lithuanian and Russian athletes [1]. Such results can be clearly related with differences among social, cultural and even structural or political structures in each country. Recently, Dimoula, Torregrosa, Psychountaki and Fernandez [5] through the comparison between Greek and Spanish elite athletes proposed a Southern European perspective of athletic career termination. It was revealed that the Southern European perspective could be characterized by voluntary career termination, lack of career termination planning, high athletic identity and relocation in the sports world. Similar results, reinforcing such idea, were observed with retired Italian football players, except for the voluntary career termination. Almost all of the Italian players referred involuntary career termination [17].

Despite of the solid results obtained in the cross-cultural perspective and for instance on the proposal of the Southern European perspective of career termination, such studies only considered how the Bronfenbrenner’s macrosystem influences the process of career termination and neglected how changes on the mesosystem (e.g., club opportunities, school) or in the microsystem (e.g., family, team, the coach, the training process, the facilities of the club) could constraint the career termination of athletes. Thus, there is a need to improve the understanding of changes at meso and micro level that characterizes and constraint the process of career termination in a specific country. Such ideas are aligned with the proposals for development of career programs in which it was clearly pointed the need to adjust such programs to each athlete specificity (e.g., individual options, type of sport, club and family characteristics) [1]. According to that, a call for a more specific socio-culturally situated approach focused on Bronfenbrenner’s meso and micro systems will allow a greater understanding of the factors that constraint career termination and to develops more adjusted local programs to former athletes [10]. Such analysis could be extremely important on the analysis of the available resources of career termination, namely the level of formal education, coping strategies or psychological support. Particularly, the level and type of coping process and the psychological support, that can be constraint by Bronfenbrenner’s meso and micro systems, should be considered as key factors for the process of successful transition [1]. In view of the above, the purpose of this study was to explore in a specific country the process of career termination of elite football players in the last 30 years, comparing the quality of career termination and the resources for career termination over the three decades of this temporal period. Such analysis will allow to understand if changes on resources for career termination at meso (e.g., club opportunities to start the practice or the school frequency), and micro level (e.g., the support of family, club or institutional support programs, the resources for transition) constraint the quality of career termination. Due to the evolution observed in last 30 years in the education, sport conditions and on the structures of the football clubs, we expect that players that retired recently reveal higher resources for career termination, allowing a better quality on career termination.

## 2. Materials and Methods

Ninety male former elite Portuguese soccer players participated in this study (M = 50.68 ± 9.14 age). Three criteria were used for selection: (a) professional male football Portuguese players; (b) participation of the national team as senior football players, and (b) career termination from football between 1985 and 2015. Considering the two criteria, a balanced sample from the retired elite Portuguese soccer players in three last decades was defined: Group I (G I) retired between 1985–1995 (*n* = 30), Group II (G II) retired between 1996–2005 (*n* = 30), and Group III (G II) retired between 2006–2015 (*n* = 30). 

The participants were informed that the information reported is confidential and only be used to research. All the participants were informed of the purpose of the study and a written informed consent was obtained. The study protocol followed the guidelines stated in the Declaration of Helsinki and was approved by the University Ethics Committee.

According to athletic career termination model (Taylor & Ogilvie, 1994) and based on the Retirement from Sports Survey [3] a questionnaire was developed for comparison between players of different decades. The questionnaire structure has been validated to ensure the reliability of the collected data. Final questionnaire was developed after exploring different opinions of previous drafts of the transcript using the following steps: (a) Adaptation of first draft of the questionnaire based on previous studies; (b) Evaluation and adjustments on the questionnaire by two senior researchers in sports sciences, who have substantial experience with questionnaires; (c) Development of a pilot study conducted with three former professional football players (d) Minor fixes and adaptations resulting from the pilot study; (f) Definition of the final version of the questionnaire. The questionnaire was defined by four sections: (a) biographical data, (b) athletic career, (c) quality of career termination and (d) available resources at the moment of career termination. In the section of biographical data, the participants reported the age, and the year of career termination. In the section of athletic career, the players reported the age when retired, the length of athletic career (i.e., the years as professional footballers) and the years as youth players (i.e., the years of formal practice between U9 to U20). In the section of quality of career transition, the participants reported the causes of career termination (participants were asked about the reasons for career termination) and the quality of the career termination (participants were asked about the level of difficulty of career termination). Regarding the reasons for career termination, aligned with previous research [7] the participants were asked to answer one of the possible reason (free choice, age, deselection, or injurie). The quality of career termination was evaluated by a multiple-choice question with three answers (“1-Easy and healthy”, “2-Regular with emotional stability” and “3-Difficult with negative emotions”).

In the section of available resources of career transition, the participants reported the level of formal education (participants were asked about the level of formal education from the options primary, middle, secondary and high level education), the coping strategies (with the identification of the activity that help them in the moment of career termination were chosen from the following possibilities: sport-related activities, social activities and foundations, family, entrepreneurship activities, job planning, political activities, and academic education), the psychological support (participants were asked about who supported them psychologically from the following possibilities: family, friends, coaches, sport agents and clubs’ presidents) and the existence of a pre-career termination plan (participants were asked about the existence of a career termination plan through a multiple-choice question with two answers). The questionnaires were administrated to the athletes in a calm environment (usually in a hotel meeting room) after a retrospective interview about their process of career termination.

Data from questionnaires were grouped into three distinct groups according to the decade of career termination of participants (G I [1985;1995], G II [1995;2005], and G III [2005;2015]). Due to the characteristics of data, the comparison of the athletic career between the three generations of retired elite football players were calculated using a one-way ANOVA, and the quality of athletic career termination and the available resources using a χ^2^ analysis. Analysis were performed with SPSS 22.0 for windows. All statistical hypothesis testing was two-tailed and a value of *p* < 0.05 was considered statistically significant.

## 3. Results

### 3.1. Athletic Career

No differences were observed between participants of different groups on age when retired (*p* > 0.05) (Table 1). Otherwise, the participants differ significantly in years in professional football and years as youth players (*p* < 0.05), with the G I revealing a higher number of years as professional footballs than G III. In opposition, G III revealed a higher number of years as youth football players than GI.

### 3.2. Quality of Career Termination

#### 3.2.1. Causes of Career Termination

No differences were observed between participants of different groups on the causes of career termination (*p* > 0.05). Free choice was the most reported cause for career termination by G I, while for G II was age and for G III was injuries. Deselection was the less frequent reported factor (10%) across all three generations (see Table 2).

#### 3.2.2. Quality of Career Termination

No differences were observed between groups on the quality of career termination (*p* > 0.05). Most of participants from all the groups reported a difficult career termination (see Table 2).

### 3.3. Available Resources

#### 3.3.1. Level of Formal Education

Significant differences were observed between groups in the level of formal education when retired (*p* < 0.05). It was observed a decrease in the number of players that referred primary education and an increase in the number of players that referred higher level education from G I to G III (see Table 3). Most of participants across all three groups revealed high school as their highest level of education.

#### 3.3.2. Coping Strategies 

No differences were observed between participants of different groups on the coping strategies when retired (*p* > 0.05). Results revealed that most of participants across all three generations were focused on sport activities, social activities and foundations and family (see Table 3). 

#### 3.3.3. Psychological Support

No differences were observed between participants of different groups on the psychological support when retired (*p* > 0.05). The family was the most important counseling reference, followed by friends and coaches (see Table 3).

#### 3.3.4. Pre-Career Termination Planning

No differences were observed between participants of different groups on pre-career termination planning. Most participants, independently of the generation context, reported the absence of plan of career termination (*p* > 0.05) (see Table 3).

## 4. Discussion

In line with the ecological model of human development, the aim of this study was to explore the process of career termination in elite Portuguese football players, comparing the quality of career termination and the resources for career termination over the last three decades. The results partially confirmed our expectations. Actually, the players that recently terminated their careers revealed higher level of formal education in comparison with others, however without implications for the quality of career termination. Remarkably, older players revealed higher length of athletic career while the recently retired players revealed longer years as youth football players. Although not analyzed here, it seems that the increase in the number of years as youth players revealing some changes on the clubs’ perspective, on the training process, and on the facilities of the clubs to improve the development of players (micro) and the increase on the higher level of formal education revealed changes on the relation between families, clubs and the school (meso system) [16]. Still, those changes didn’t contribute to improve the quality of career termination of elite Portuguese football players. Further research is required to analyze not only the players’ perspective regarding career paths and career retirement but also the perspective of clubs, schools and families allowing a clear understanding of the impacts of changes on micro and meso systems on the career termination of football players. That is, further studies should be developed with new and validated questionnaires or interviews that clearly evaluate the Bronfenbrenner’s meso and micro systems.

The results seem to follow the common characteristics of sport career termination in other European cultures [5]. That is to say, professional football players reported a difficult process to adjust their life after their athletic career termination. Such result can be linked with the great number of players that refer injuries, deselection as well as age as the main causes for career retirement, factors associated with the difficulties in adaptation to the new life [3,5,18]. Despite the difficulties on athletic career termination reported, it was observed an increase over the decades in the level of formal education. In last decades, formal education increased, and a higher number of players were enrolled in higher-level education. Surprisingly, the increase in the educational level did not contribute to improve the quality of career termination [7]. Thus, more than to be constrained by the surrounding environment, the quality of the process of career termination can be explained by the labor market and/or the low level of players’ resources. The low preparation and feeling of poor control on the process of career termination felt when injuries, deselection and, in some cases, age were reported contributed difficulties into the new life style transition [4].

These Portuguese football players reported, over those decades, similar coping strategies to deal with career termination, including time dedicated either to sport related activities or to family and social support activities, social activities and foundations that might be categorized as a productive and positive response according to several authors (Alfermann et al., 2004; Wylleman, Alfermann, & Lavallee, 1999). Also, an available resource to face career termination and social adjustment is the development of support from family and friends [1].

Consistent with the results obtained in other sports, it was observed that the players for all the decades did not have, in general, a plan for career termination. Captivatingly, previous research reported that the plan for career termination had higher influence on the quality of career transition than the nationalities or cultural differences [3,10]. Our results highlight that the lack of plans for career termination had higher influence on the quality of career transition than the available resources of players such as the level of formal education per se. This result helps to clarify why the quality of career termination was, in general, reported as difficult over the last three decades, despite of the increase in the level of education or the number of years as youth players [16]. As previously pointed, the athlete career termination is a process in which the micro, meso, and macro levels contributes to enrich and prepare the players to develop better feelings and strengthen their level of control over the process. Thus, there is a need to create a transversal culture of management of careers that should be part of everyday life of elite athletes [15]. The development of programs of dual careers and plans of career termination supported by all the agents involved in the process (schools, clubs, sport federations, leagues, career managers, sponsors and governments) should engage the players and improve their ability to manage their career. This is crucial to improve the quality of career termination of professional football players [1]. Further research should be developed to better understand the levels of correlation between the quality of career termination and each of their available resources.

Although the current research presents some limitations due to the used questionnaire, the analysis and comparison between the three last generations of elite players that ended their athlete career help to understand that, despite of the great differences observed in general society, the problems of career termination remain the same. Career retirement are difficult processes that need to be prepared over the entire career and the existence of supportive programs are paramount to improve the preparedness and the level of voluntariness of elite players for career termination. 

## 5. Conclusions

The main purpose of the current study was to explore the process of career termination of elite football players in the last 30 years, comparing the quality of career termination and the resources for career termination over those three decades. The results highlighted similarities on career termination over the last three decades and revealed similarities with the athletic career termination of athletes from Southern European cultures. 

Despite the evolution in the level of the education of the former athletes for the last three decades, athletic career termination remains difficult because it almost doesn’t exist any plan for that life period transition. In such circumstances and considering the huge socioeconomic relevance of football, it must be a priority, for several authorities and sport organizations, the development of integrated support programs for athletes, enabling them to plan their career termination in a qualified and sustainable manner.

## Figures and Tables

**Table 1:** Means (and standard deviations) for participants’ characteristics.

Variables	G I	G II	G III	*F*	*p*
Age when retired	36.13 (4.05)	35.6 (2.53)	34.86 (4.15)	0.91	0.406
Years in professional football	17.07 (2.52)	16.13 (2.40)	14.8 (3.88) *	4.30	0.017
Years as youth player	3.53 (1.40)	5.53 (1.88) ^#^	7.77 (1.30) *	55.66	0.000

Note: G I [1985;1995]; G II [1995;2005]; G III [2005;2015]. * Significant difference (*p* < 0.05) between G I and G III; ^#^ Significant difference (*p* < 0.05) between G I and G II.

**Table 2:** Analysis of quality of career termination: causes and quality of career termination.

Variable	G I		G II		G III		*χ* ^2^	*p*
	F	%	F	%	F	%		
**Causes of career termination**								
Free choice	10	33.3	10	33.3	9	30	4.075	0.66
Age	8	26.7	12	40.0	7	23.3		
Injuries	8	26.7	5	16.7	11	36.7		
Deselection	3	10	3	10.0	3	10.0		
**Quality of career termination**								
Easy	8	26.7	9	30	7	23.3	2.404	0.62
Regular	6	20	8	26.7	11	36.7		
Difficult	16	53.3	13	43.3	12	40		

Note: G I [1985;1995]; G II [1995;2005]; G III [2005;2015].

**Table 3:** Analysis of available resources: level of formal education, coping strategies, psychological support and pre-career termination planning.

Variable	G I		G II		G III		*χ* ^2^	*p*
	F	%	F	%	F	%		
**Level of formal education**								
Primary education	5	16.7	-	-	-	-	12.781	0.04
Middle education	9	30.0	8	26.7	7	23.3		
Secondary education	14	46.7	18	60.0	17	56.7		
High-level education	2	6.7	4	13.3	6	20.0		
**Coping strategies**								
Sport related activities	10	33.3	9	30.0	6	20.0	2.404	0.62
Social activities and foundations	3	10.0	6	20.0	8	26.7		
Family	4	13.3	8	26.7	6	20,0		
Entrepreneurship activities	8	26.7	4	13.3	3	10.0		
Job planning	5	16.7	1	3.3	5	16.7		
Political activities	-	-	1	3.3	1	3.3		
Academic education	-	-	1	3.3	1	3.3		
**Psychological support**								
Family	16	53.3	15	50.0	14	46.7	3.66	0.89
Friends	7	23.3	6	20.0	6	20.0		
Coaches	6	20.0	6	20.0	5	16.7		
Sport agents	-	-	2	6.7	3	10.0		
Clubs’ presidents	1	3.3	1	3.3	2	6.7		
**Pre-career termination planning**								
No	24	80	26	86.7	25	83.3	3.66	0.89
Yes	6	20	4	13.3	5	16.7		

Note: G I [1985;1995]; G II [1995;2005]; G III [2005;2015].
